# Importance of Medical Journals

**Published:** 1879-04-20

**Authors:** 


### *IMPORTANCE OF MEDICAL JOURNALS.*

It is certainly a lamentable fact that a very large proportion of those engaged in the practice of medicine, neither take nor read Medical Journals. The result is that they make no progress in the professson, deprive themselves of important and essential information, and subject their patients to needless suffering, and sometimes to *unnecessary death.* We say unnecessary, because of the ignorance of agents, or methods which might have saved life, if the practitioner had known of their existence.

It may be charity to suppose that some do not realize the terrible responsibility of their negligence, that others are too poor to pay for a Medical Journal. When we consider the exceeding cheapness of medical literature in these days, and the fact that the charge for a single visit and prescription would cover the expense of a good journal for a whole year, it is hard to accept this last excuse. Yet we have done all that we could to accommodate our journal to the necessities of our medical brethren. Knowing the pressure that has been upon the profession since the war, and that a majority of practitioners feel themselves unable to take more than one Journal, we have endeavored to find that medium as to size and style that would, as nearly as could be, cover the entire field of medical news, at the lowest possible cost to the subscriber.

It is believed that our Journal more nearly conforms to these conditions—is more practical in its plan and objects, and better adapted to the wants of the busy practitioner than any Journal which has heretofore existed in the United States. Though not large or extravagant in style, its table of contents exhibits a review of the entire field of Medical Literature. Its plain, practical and condensed method, by which the information given may be made available at the bed side, renders it particularly acceptable to the busy practitioner; while its low price of subscription accords well with the stringency of the times.

THE SOUTHERN MEDICAL RECORD was established in 1870. That it has become the favorite of the busy practitioner, we have hundreds of testimonials to show, in the correspondence of the office. And we feel assured that if we could personally visit and present these facts to medical men, that thousands of them would take our Journal, who are now indifferent on the subject. It would seem indeed that no argument would be required to convince any physician that duty to his own conscience, not less than those who trust their lives in his hands, requires him to keep posted in the progress of his profession, and this he cannot do, without taking at least one medical Journal.

We earnestly hope that every one who reads these lines will appreciate the truth of the above remarks, and at once subscribe for our Journal; not only so, but that he will induce others to subscribe, and aid us by his pen and influence in our work of developing the medical literature, and advancing the progress of medical science in our section.

